# Misdiagnosis of scrub typhus as hemorrhagic fever with renal syndrome and potential co-infection of both diseases in patients in Shandong Province, China, 2013–2014

**DOI:** 10.1371/journal.pntd.0009270

**Published:** 2021-03-30

**Authors:** Xiao-lan Gu, Rui Qi, Wen-qian Li, Yong-jun Jiao, Hao Yu, Xue-jie Yu

**Affiliations:** 1 State Key Laboratory of Virology, School of Health Sciences, Wuhan University, Wuhan, China; 2 Institute of Pathogenic Microbiology, Jiangsu Provincial Center for Disease Prevention and Control, Key Laboratory of Enteric Pathogenic Microbiology, Ministry Health, Nanjing, China; Chengde Medical University, CHINA

## Abstract

**Background:**

Scrub typhus, caused by *Orientia tsutsugamushi*, an obligate intracellular gram-negative bacterium, along with hemorrhagic fever with renal syndrome (HFRS), caused by hantaviruses, are natural-focus infectious diseases prevalent in Shandong Province, China. Both diseases have similar clinical manifestations in certain disease stages and similar epidemic seasons, which has caused difficulties for physicians in distinguishing them. The aim of this study was to investigate whether misdiagnosis of scrub typhus as HFRS occurred in patients in Shandong Province.

**Methods:**

Serum samples (N = 112) of clinically suspected HFRS patients from 2013 to 2014 in Shandong Province were analyzed with enzyme-linked immunosorbent assay (ELISA) for antibodies to both hantavirus and *Orientia tsutsugamushi*.

**Results:**

ELISA showed that 56.3% (63/112) and 8.0% (9/112) of clinically suspected HFRS patients were IgM antibody positive to hantavirus and *O*. *tsutsugamushi*, respectively. Among the hantavirus IgM antibody positive patients, 7.9% (5/63) were also IgM antibody positive to *O*. *tsutsugamushi*. Among the hantavirus IgM antibody negative sera, 8.2% (4/49) of sera were positive to *O*. *tsutsugamushi*.

**Conclusions:**

We concluded that some scrub typhus patients were misdiagnosed as HFRS and co-infection of scrub typhus and HFRS might exist in China. Due to the different treatments for scrub typhus and HFRS, physicians should carefully differentiate between scrub typhus and HFRS and consider administering anti-rickettsia antibiotics if treatment for HFRS alone does not work.

## Author summary

Scrub typhus is caused by *O*. *tsutsugamushi* and hemorrhagic fever with renal syndrome (HFRS) is caused by hantavirus. Both diseases have similar manifestations and have their peak epidemic season in the fall, which makes their clinical differentiation difficult to be differentiated from each other clinically. In this study, we found that scrub typhus was indeed misdiagnosed as HFRS and co-infection of *O*. *tsutsugamushi* and hantaviruses might exist in China. Owing to different treatments of HFRS and scrub typhus, physicians need pay attention to differentiate them; identify co-infection of HFRS and scrub typhus; and consider administering antibiotics if antiviral treatment alone does not work.

## Introduction

Scrub typhus, also known as tsutsugamushi disease, is an acute febrile infectious disease that is caused by *Orientia tsutsugamushi*, an obligate intracellular gram-negative bacterium [1]. The infection is transmitted to humans or rodents primarily by the bite of chigger mites [2]. Typical presentations for scrub typhus include fever and skin eschars, with fever presenting before other symptoms. However, skin eschars are easily neglected on examination and tracing any patient history of chigger bites is also difficult. Therefore, scrub typhus is often misdiagnosed as other febrile illnesses [3]. The disease has been traditionally reported in the Tsutsugamushi Triangle in the Asia-Pacific region, but the disease has emerged in non-traditional areas in recent years [4].

Hemorrhagic fever with renal syndrome (HFRS) is a natural-focus infectious disease caused by hantaviruses, mainly Hantaan orthohantavirus and Seoul orthohantavirus, members of the *Hantaviridae* family, and are primarily transmitted by rodents [5, 6]. The disease was thought to be transmitted to humans by inhaling aerosolized excreta of infected animals, or through direct contact with infected rodents and their feces, saliva or blood [7]. The major clinical manifestations of HFRS include fever, hemorrhage and renal failure [8]. HFRS is widely distributed in the world with China reporting the highest incidence [9].

Based on the fact that scrub typhus and HFRS have similar clinical features during the early phase with atypical and indistinguishable febrile symptoms; Shandong Province of China is endemic for both scrub typhus and HFRS; and both diseases have their peak epidemic season in the fall in China [10–13], we reason that the two diseases may be misdiagnosed with each other. Therefore, we investigated whether scrub typhus was misdiagnosed as HFRS.

## Methods

### Ethics statement and consent to participate

Ethical approval of this study was admitted by the ethics committees of Wuhan University (2018010). The data are anonymous, and informed consents were not obtained.

### Sample source and study design

Acute-phase serum samples of clinically suspected HFRS patients from 2013 to 2014 were collected from local centers for disease control and prevention (CDC) from Zibo and Qingdao cities in Shandong Province, China and were stored at -80°C. The landscape of Zibo City is mainly mountains (42.0%) and hills (29.9%) and Qingdao is a city with seashores and hills. Blood samples of clinically suspected HFRS patients were collected in hospitals and were routinely submitted to the local CDC. We collected patients’ blood samples and medical records, including basic information, clinical manifestations and laboratory tests from the local CDC. Clinical suspected cases of HFRS were diagnosed by local physicians following the criteria of the Chinese CDC (http://www.chinacdc.cn/did/crbzt/dwyxhmjcrb/lxxcxrx/). The IgM positive samples were considered as confirmed cases for HFRS or scrub typhus in this study. The criteria for suspected and confirmed cases of HFRS were described in Table 1.

**Table 1:** Case definitions of HFRS.

Case classification	Case definitions
**Clinically suspected cases**	Had epidemiological history, symptoms and/or signs (acute onset, chills, febrile, fatigue; headache, orbital pain, low back pain; face, neck, and upper chest congestion and flushing, showing drunken appearance; conjunctival congestion; bleeding points; tourniquet test positive) in the early stage of the disease
**Laboratory confirmed cases**	Suspected cases plus any of the following characteristics (IgM antibodies were positive to hantaviruses, IgG antibodies in convalescent serum experienced a 4-fold or greater rise than that in acute-phase serum, or hantavirus and/or hantavirus RNA was detected and/or isolated from the serum of patients)

### Detection of IgM antibody

Acute-phase sera of 112 clinically suspected HFRS patients were retrospectively tested for IgM antibodies against *O*. *tsutsugamushi* in this study and IgM antibodies against hantavirus had been detected by us previously [14] using double-antigen sandwich ELISA. *Orientia tsutsugamushi* IgM antibodies were detected with Diagnostic Kit for Human IgM Detection of Tsutsugamushi Diseases from Wending Biotech (Nanjing, Jiangsu, China). The 96-well plates were coated with purified anti-human IgM (μ-chain monoclonal antibody) to specifically capture IgM antibodies in the serum, and captured IgM antibodies against *O*. *tsutsugamushi* were detected with HRP-conjugated 56KDa membrane proteins of *O*. *tsutsugamushi*. Hantavirus IgM antibodies were detected with HFRS-IgM ELISA Kit (Wantai Biological Pharmacy, Beijing, China) according to the instruction of the manufacturer. Undiluted serum was tested for IgM antibodies against hantavirus and diluted serum was tested for IgM antibodies against *O*. *tsutsugamushi*. The optical density (OD) value was measured at 450 nm using a plate reader. Cutoff value was set through formula suggested by the instructions of both ELISA kits (cutoff value for *O*. *tsutsugamushi* detection = 0.748 × average OD value of the negative control + 0.146, cutoff value for hantavirus detection = average OD value of the negative control + 0.1). The negative control reagents were provided by ELISA kits. A sample was considered seropositive if the OD value was greater than the cutoff value.

## Results

### Serological study

**Fig 1:**
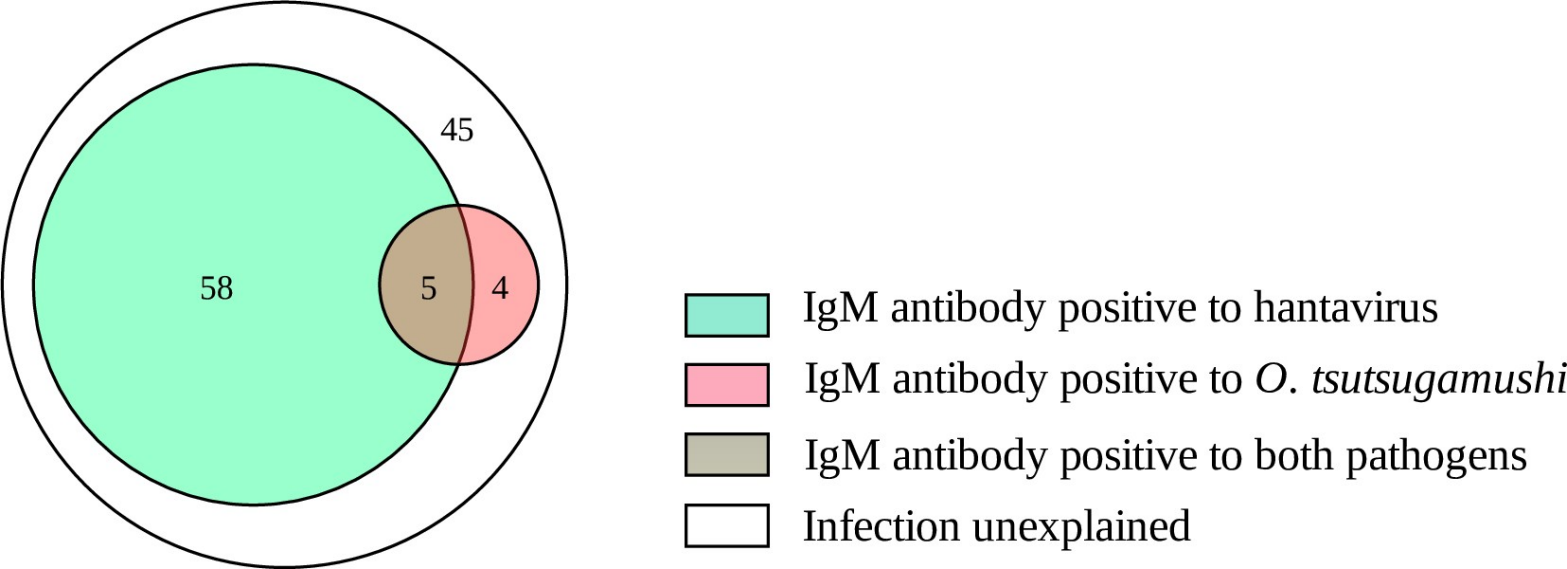
Venn diagram indicates the relationship among clinically suspected HFRS patients.

**Fig 2:**
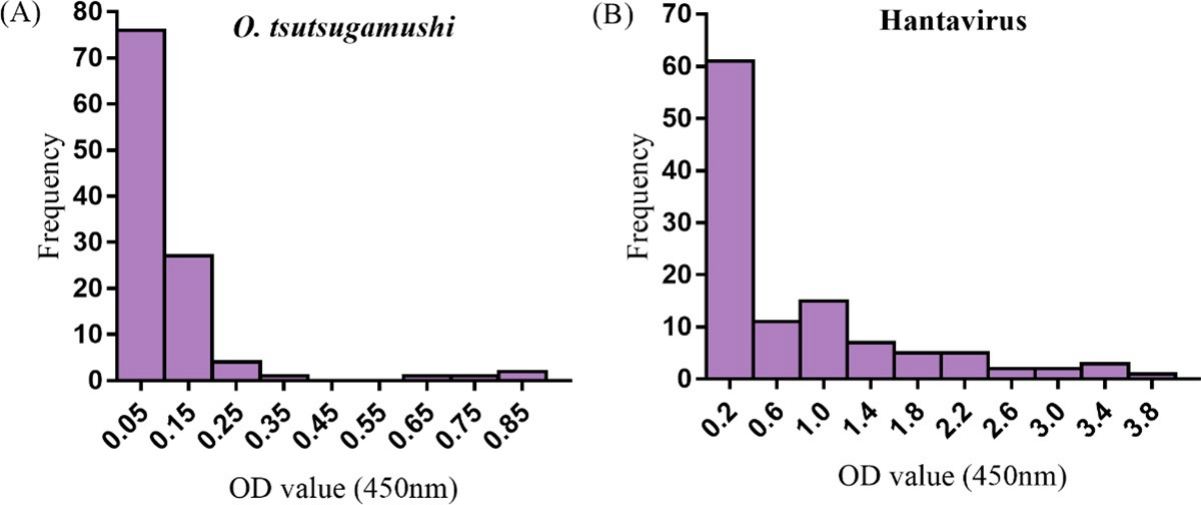
Frequency distribution of the OD values in ELISA. (A) Distribution of the OD values for IgM antibody to *O*. *tsutsugamushi*. Cut-off: 0.1960. (B) Distribution of the OD values for IgM antibody to hantavirus. Cut-off: 0.1753.

ELISA results showed that among 112 clinically suspected HFRS patients, 63 (56.3%) were IgM antibody positive to hantavirus and 9 (8.0%) were IgM antibody positive to *O*. *tsutsugamushi*. Among the confirmed HFRS cases, 7.9% (5/63) of patients were also IgM antibody positive to *O*. *tsutsugamushi*. Among the 49 hantavirus IgM antibody negative sera, 8.2% (4/49) were positive to *O*. *tsutsugamushi* (Figs 1 and 2).

### Characteristics of patients

Among 112 clinically suspected HFRS patients, 101 had clinical data. The demographic characteristics, epidemiological features, clinical features and laboratory data of patients with scrub typhus, HFRS, co-infection, and unexplained febrile illness were summarized in Table 2.

**Table 2:** Clinical characteristics and laboratory diagnosis of 101 suspected HFRS patients.

Characteristics	Co-infection patients ^a^	Scrub typhus patients	HFRS patients	Unexplained patients
	(N = 5)	(N = 3)	(N = 55)	(N = 38)
**Demographics**				
Median age (range)	49 (42–61)	59 (41–66)	47 (15–80)	53 (21–79)
Male/female	3/2	2/1	33/22	23/15
Famers	5 (100%)	3 (100%)	52 (96%)	30 (79%)
Rural area	5 (100%)	3 (100%)	51 (93%)	30 (79%)
**Epidemiological features**				
Contact with rodents 1 month before onset	0 (0%)	2 (25%)	13 (22%)	7 (18%)
Rodents or excrement near the house	4 (80%)	2 (67%)	30 (55%)	26 (68%)
**Clinical features**				
Acute onset	4 (80%)	2 (67%)	48 (87%)	34 (89%)
Fatigue	4 (80%)	2 (67%)	41 (75%)	30 (79%)
Fever	5 (100%)	2 (67%)	55 (100%)	36 (95%)
Headache	4 (80%)	1 (33%)	42 (76%)	25 (66%)
Low back pain	4 (80%)	2 (67%)	36 (65%)	23 (61%)
Orbital pain	1 (20%)	1 (33%)	20 (36%)	16 (42%)
Flushed face	3 (100%)	1 (33%)	32 (58%)	16 (42%)
Flushed neck	3 (60%)	1 (33%)	18 (33%)	12 (32%)
Flushed chest	2 (40%)	1 (33%)	14 (25%)	10 (26%)
Bleeding point	0 (0%)	0 (0%)	8 (15%)	0 (0%)
Hematuria	0 (0%)	0 (0%)	14 (25%)	3 (8%)
**Laboratory examination**				
Leukopenia ^b^	1 (20%)	0 (0%)	12 (22%)	10 (26%)
Leukocytosis ^c^	1 (20%)	1 (33%)	9 (16%)	14 (37%)
Thrombocytopenia ^d^	4 (80%)	3 (100%)	47 (85%)	26 (68%)
Proteinuria ^e^	2 (40%)	2 (67%)	50 (91%)	8 (21%)

^a^ Co-infection: patients were IgM positive to both Orientia tsutsugamushi and hantavirus antigens.

^b^ Leukopenia: leukocyte count<4×10^3^/μl.

^c^ Leukocytosis: leukocyte count>11×10^3^/μl.

^d^ Thrombocytopenia: platelet count<50×10^3^/μl.

^e^ Proteinuria: 24-hour urinary protein > 150mg.

## Discussion

In this study we showed that some clinically suspected HFRS patients were IgM antibody positive to *O*. *tsutsugamushi*, but were negative to hantavirus. In a previous study we had analyzed serum antibody of severe fever with thrombocytopenia syndrome virus (SFTSV) with ELISA in the same sera of clinically suspected HFRS patients used in this study and demonstrated that 4 patients were IgM antibody positive to SFTSV, but negative to hantavirus [14]. Our studies indicated that both scrub typhus and SFTSV infection could be misdiagnosed as HFRS in China. Our studies also indicated that HFRS is often blamed as the culprit of many infectious diseases in Shandong Province of China, because Shandong Province is one of the most serious endemic areas of HFRS, scrub typhus and severe fever with thrombocytopenia syndrome (SFTS) [101113–15]. This led to physicians commonly diagnosing febrile patients with atypical symptoms as HFRS. Because all three aforementioned diseases are prevalent in Shandong Province [15–17], the possibility of misdiagnosis and co-infection cannot be ignored, and further epidemiological investigation is needed. Except for confirmed scrub typhus, SFTS and HFRS cases, a considerable number (n = 45, 35%) of patients were still unconfirmed although they were clinically diagnosed as HFRS. Although we do not know what disease these patients actually had, we cannot exclude that some of the patients could be have had HFRS, scrub typhus, or SFTS due to a lack of convalescent sera of the patients to confirm the diagnosis. Some of them could experience human granulocytic anaplasmosis [18], Japanese spotted fever [19] or leptospirosis [20] according to their undistinguished clinical manifestations, and although the epidemic seasons of these do not overlap traditionally, the season of disease occurrence can vary with climate change and environmental factors.

We also found that some patients were IgM antibody positive to both *O*. *tsutsugamushi* and hantaviruses. One possibility is that these patients were co-infected with both hantavirus and *O*. *tsutsugamushi* at the same time. However, serum samples had been frozen and thawed several times, and due to the poor quality of the samples and their volume limitations, we were unable to perform PCR/RT-PCR on the samples to confirm whether they were co-infected with both *O*. *tsutsugamushi* and hantaviruses from pathogens. Another possibility is that these patients were sequentially infected with *O*. *tsutsugamushi* and hantavirus in a short period as IgM antibodies might persist for several months. Therefore, the co-infection needs to be further studied by isolation of the pathogens or detection of their nucleic acids in single patients in the future.

Based on the available clinical data of 3 scrub typhus patients, all scrub typhus patients had nonspecific and similar symptoms with HFRS patients, and none of the 3 scrub typhus patients had pin-point bleeding or hematuria. The prominent clinical features of HFRS patients included fever (n = 55, 100%), proteinuria (n = 50, 91%), acute onset (n = 48, 87%) and thrombocytopenia (n = 47, 85%), which is consistent with the previous study [21]. We noticed that some HFRS patients showed typical symptoms of ache (headache, low back pain and orbital pain) and redness (flushed neck, flushed face and flushed chest), which were similarly exhibited by some scrub typhus patients. In a study, it was found that facial flushing was also presented in 62% scrub typhus patients [12], therefore, facial flushing is not a unique feature for HFRS patients. Other typical symptoms such as fever and proteinuria of HFRS patients [14] were also observed in two scrub typhus patients. Furthermore, while it has been reported that thrombocytopenia was a general and outstanding feature in all enrolled HFRS patients [22], our study found 3 patients who were only infected with scrub typhus that also had thrombocytopenia. A previous report indicated that more than 73% (166/259) of scrub typhus patients had thrombocytopenia [23]. HFRS patients with antibodies to both hantavirus and *O*. *tsutsugamushi* had similar clinical manifestations with scrub typhus patients or HFRS patients. These undifferentiated manifestations made it more difficult for physicians to diagnose scrub typhus, HFRS and co-infections. If the physician only carries out antiviral therapy, it is possible to aggravate the degree of damage done to the body of scrub typhus patients and may result in their death by multi-organ failure [24]. The 3 confirmed scrub typhus patients, 96% of HFRS patients and 5 co-infection patients were all farmers. Farmers work in fields, allowing them more opportunities of being exposed to infected rodents, which may carry or harbor both *O*. *tsutsugamushi* [25] and hantavirus [26]. This is consistent with the conclusion that farmers constitute the majority of cases of scrub typhus and HFRS [27, 28].

Antibody detection is a simple and rapid diagnostic method compared with pathogen isolation and detection of nucleic acid. However, due to the limitations of the ELISA and non-specific reaction, there may be false positive and false negative results in actual detection. In our study, the upper and lower area of the cutoff value were set as the gray area, on the basis of kit instructions, and the OD value of samples falling in the gray area were re-detected to obtain accurate results, which was directly related to the diagnosis. The sensitivity of the IgM-ELISA kit for HFRS and scrub typhus were 98.2% and 95%, and the specificity of that were 99.9% and 100.0%, respectively, according to the kit manufacturers. In addition, the conserved region of 56KDa protein of *O*. *tsutsugamushi* covered Gilliam, Karp, Kato, Kawasaki and other serotypes was expressed and purified, then conjugated by HRP and used as the diagnostic antigen. The kit manufacture declared that the IgM-ELISA kit for scrub typhus had no cross reaction with serum of other undistinguished diseases, such as Japanese spotted fever and SFTS, so the positive case of scrub typhus is certain, confirming our hypothesis that existing misdiagnosis of HFRS and coinfection with scrub typhus. Furthermore, serum samples were collected during the acute phase of the clinically suspected HFRS patients, and is suitable for retrospective diagnosis for scrub typhus with IgM ELISAs [29]. These evidences demonstrated that the confirmed diagnosis of scrub typhus patients was reliable.

As we have found in previous publication utilizing the same sample set, there was a misdiagnosis of SFTS among these clinically-diagnosed HFRS patients [14], this time with potential co-infection by scrub typhus. When multiple diseases exist within a defined geographic area, and have similar clinical features, similar or overlapping seasons of occurrence, and similar risk factors (e.g., outdoor activities), misdiagnosis or co-infection is inevitable. If patients were diagnosed without pathogen-specific assays like 16S rRNA PCR or RT-PCR for confirmation of diagnosis, this problem will be amplified not just with scrub typhus and HFRS, but also with SFTSV. Therefore, physicians should pay attention to pathogenic examination, perform relevant examinations in a timely manner, evaluate the clinical value of laboratory examinations objectively, and antiviral drugs should be combined with antibiotics when necessary.

In summary, our study indicated that scrub typhus could be misdiagnosed as HFRS and dual infection of scrub typhus and HFRS may exist in Shandong Province. These results will call physicians’ attention, not only in Shandong Province but also in other endemic regions where both scrub typhus and HFRS are prevalent, to improve their ability and method in differentiating between scrub typhus and HFRS and to note that HFRS patients may be co-infected with scrub typhus while also considering administering anti-rickettsia antibiotics if treatment for HFRS alone does not work.

## References

[1] Giengkam S, Blakes A, Utsahajit P, Chaemchuen S, Atwal S, Blacksell SD, et al. Improved quantification, propagation, purification and storage of the obligate intracellular human pathogen *Orientia tsutsugamushi*. PLoS Negl Trop Dis, 2015. 9(8): p. e0004009. 10.1371/journal.pntd.0004009 26317517 PMC4552649

[2] Walker DH. Scrub typhus—scientific neglect, ever-widening impact. N Engl J Med, 2016. 375(10): p. 913–5. 10.1056/NEJMp1608499 27602663

[3] Park SW, Ha NY, Ryu B, Bang JH, Song H, Kim Y, et al. Urbanization of scrub typhus disease in South Korea. PLoS Negl Trop Dis, 2015. 9(5): p. e0003814. 10.1371/journal.pntd.0003814 26000454 PMC4441427

[4] Weitzel T, Dittrich S, Lopez J, Phuklia W, Martinez-Valdebenito C, Velasquez K, et al. Endemic scrub typhus in South America. N Engl J Med, 2016. 375(10): p. 954–61. 10.1056/NEJMoa1603657 27602667

[5] Kuhn JH, Adkins S, Alioto D, Alkhovsky SV, Amarasinghe GK, Anthony SJ, et al. 2020 taxonomic update for phylum Negarnaviricota (Riboviria: Orthornavirae), including the large orders Bunyavirales and Mononegavirales. Arch Virol, 2020. 165(12): p. 3023–72. 10.1007/s00705-020-04731-2 32888050 PMC7606449

[6] Fang LZ, Zhao L, Wen HL, Zhang ZT, Liu JW, He ST, et al. Reservoir host expansion of hantavirus, China. Emerg Infect Dis, 2015. 21(1): p. 170–1. 10.3201/eid2101.140960 25531113 PMC4285249

[7] Fang LQ, Zhao WJ, de Vlas SJ, Zhang WY, Liang S, Looma CW, et al. Spatiotemporal dynamics of hemorrhagic fever with renal syndrome, Beijing, People’s Republic of China. Emerg Infect Dis, 2009. 15(12): p. 2043–5. 10.3201/eid1512.081078 19961697 PMC3044508

[8] Zhao Y, Ge L, Zhou Y, Sun Z, Zheng E, Wang X, et al. A new seasonal difference space-time autoregressive integrated moving average (SD-STARIMA) model and spatiotemporal trend prediction analysis for hemorrhagic fever with renal syndrome (HFRS). PLoS One, 2018. 13(11): p. e0207518. 10.1371/journal.pone.0207518 30475830 PMC6261020

[9] Xiao H, Tian HY, Gao LD, Liu HN, Duan LS, Basta N, et al. Animal reservoir, natural and socioeconomic variations and the transmission of hemorrhagic fever with renal syndrome in Chenzhou, China, 2006–2010. PLoS Negl Trop Dis, 2014. 8(1): p. e2615. 10.1371/journal.pntd.0002615 24421910 PMC3888453

[10] Wang L, Wang T, Cui F, Zhai SY, Zhang L, Yang SX, et al. Hemorrhagic fever with renal syndrome, Zibo City, China, 2006–2014. Emerg Infect Dis, 2016. 22(2): p. 274–6. doi: 10.3201eid/2202.151516 26812444 10.3201/eid2202.151516 PMC4734509

[11] Jiang F, Zhang Z, Dong L, Hao B, Xue Z, Ma D, et al. Prevalence of hemorrhagic fever with renal syndrome in Qingdao City, China, 2010–2014. Sci Rep, 2016. 6: p. 36081. 10.1038/srep36081 27786303 PMC5081555

[12] Zhang S, Song H, Liu Y, Li Q, Wang Y, Wu J, et al. Scrub typhus in previously unrecognized areas of endemicity in China. J Clin Microbiol, 2010. 48(4): p. 1241–4. 10.1128/JCM.01784-09 20129967 PMC2849583

[13] Liu YX, Feng D, Suo JJ, Xing YB, Liu G, Liu LH, et al. Clinical characteristics of the autumn-winter type scrub typhus cases in south of Shandong Province, northern China. BMC Infect Dis, 2009. 9: p. 82. 10.1186/1471-2334-9-82 19493361 PMC2703643

[14] Qi R, Qin XR, Wang L, Han HJ, Cui F, Yu H, et al. Severe fever with thrombocytopenia syndrome can masquerade as hemorrhagic fever with renal syndrome. PLoS Negl Trop Dis, 2019. 13(3): p. e0007308. 10.1371/journal.pntd.0007308 30925154 PMC6457554

[15] Zhao L, Zhai SY, Wen HL, Cui F, Chi YY, Wang L, et al. Severe fever with thrombocytopenia syndrome virus, Shandong Province, China. Emerg Infect Dis, 2012. 18(6): p. 963. 10.3201/eid1806.111345 22608264 PMC3358154

[16] Zhang C, Fu X, Zhang Y, Nie C, Li L, Cao H, et al. Epidemiological and time series analysis of hemorrhagic fever with renal syndrome from 2004 to 2017 in Shandong Province, China. Sci Rep, 2019. 9(1): p. 14644. 10.1038/s41598-019-50878-7 31601887 PMC6787217

[17] Wu YC, Qian Q, Soares Magalhaes RJ, Han ZH, Hu WB, Haque U, et al. Spatiotemporal dynamics of scrub typhus transmission in mainland China, 2006–2014. PLoS Negl Trop Dis, 2016. 10(8): p. e0004875. 10.1371/journal.pntd.0004875 27479297 PMC4968795

[18] Wormser GP, Dattwyler RJ, Shapiro ED, Halperin JJ, Steere AC, Klempner MS, et al. The clinical assessment, treatment, and prevention of lyme disease, human granulocytic anaplasmosis, and babesiosis: clinical practice guidelines by the Infectious Diseases Society of America. Clin Infect Dis, 2006. 43(9): p. 1089–134. 10.1086/508667 17029130

[19] Sando E, Suzuki M, Katoh S, Fujita H, Taira M, Yaegashi M, et al. Distinguishing Japanese spotted fever and scrub typhus, Central Japan, 2004–2015. Emerg Infect Dis, 2018. 24(9): p. 1633–41. 10.3201/eid2409.171436 30124190 PMC6106405

[20] Zubach O, Telegina T, Semenyshyn O, Vasiunets L, Zinchuk A. Leptospirosis in Ukraine (Lviv Oblast): clinical and epidemiological features. Vector Borne Zoonotic Dis, 2019. 19(5): p. 341–6. 10.1089/vbz.2018.2375 30335592 PMC6486673

[21] Liu YX, Feng D, Zhang Q, Jia N, Zhao ZT, De Vlas SJ, et al. Key differentiating features between scrub typhus and hemorrhagic fever with renal syndrome in northern China. Am J Trop Med Hyg, 2007. 76(5): p. 801–5. 17488894

[22] Rista E, Pilaca A, Akshija I, Rama A, Harja E, Puca E, et al. Hemorrhagic fever with renal syndrome in Albania. Focus on predictors of acute kidney injury in HFRS. J Clin Virol, 2017. 91: p. 25–30. 10.1016/j.jcv.2017.03.021 28411480

[23] Attur RP, Kuppasamy S, Bairy M, Nagaraju SP, Pammidi NR, Kamath V, et al. Acute kidney injury in scrub typhus. Clin Exp Nephrol, 2013. 17(5): p. 725–9. 10.1007/s10157-012-0753-9 23292176

[24] Peter JV, Sudarsan TI, Prakash JA, Varghese GM. Severe scrub typhus infection: clinical features, diagnostic challenges and management. World J Crit Care Med, 2015. 4(3): p. 244–50. 10.5492/wjccm.v4.i3.244 26261776 PMC4524821

[25] Rodkvamtook W, Kuttasingkee N, Linsuwanon P, Sudsawat Y, Richards AL, Somsri M, et al. Scrub typhus outbreak in Chonburi Province, Central Thailand, 2013. Emerg Infect Dis, 2018. 24(2): p. 361–5. 10.3201/eid2402.171172 29350148 PMC5782907

[26] Xiao H, Tong X, Gao L, Hu S, Tan H, Huang ZYX, et al. Spatial heterogeneity of hemorrhagic fever with renal syndrome is driven by environmental factors and rodent community composition. PLoS Negl Trop Dis, 2018. 12(10): p. e0006881. 10.1371/journal.pntd.0006881 30356291 PMC6218101

[27] Lee HW, Cho PY, Moon SU, Na BK, Kang YJ, Sohn Y, et al. Current situation of scrub typhus in South Korea from 2001–2013. Parasit Vectors, 2015. 8: p. 238. 10.1186/s13071-015-0858-6 25928653 PMC4416255

[28] Sun Y, Wei YH, Yang Y, Ma Y, de Vlas SJ, Yao HW, et al. Rapid increase of scrub typhus incidence in Guangzhou, southern China, 2006–2014. BMC Infect Dis, 2017. 17(1): p. 13. 10.1186/s12879-016-2153-3 28056840 PMC5216553

[29] Devamani CS, Prakash JAJ, Alexander N, Suzuki M, Schmidt WP. Hospitalisations and outpatient visits for undifferentiated fever attributable to scrub typhus in rural South India: Retrospective cohort and nested case-control study. PLoS Negl Trop Dis, 2019. 13(2): p. e0007160. 10.1371/journal.pntd.0007160 30802243 PMC6405239

